# Antibacterial Activity of Lactic Acid Producing *Leuconostoc mesenteroides* QZ1178 Against Pathogenic *Gallibacterium anatis*

**DOI:** 10.3389/fvets.2021.630294

**Published:** 2021-04-22

**Authors:** Hua Zhang, HePing HuangFu, Xing Wang, ShanShan Zhao, Yuan Liu, Haoxin Lv, GuangYong Qin, Zhongfang Tan

**Affiliations:** ^1^Henan Key Laboratory of Ion-Beam Bioengineering, School of Agricultural Sciences, Zhengzhou University, Zhengzhou, China; ^2^School of Food and Biological Engineering, Henan University of Animal Husbandry and Economy, Zhengzhou, China; ^3^Henan Key Laboratory of Ion-Beam Bioengineering, School of Physics and Microelectronics, Zhengzhou University, Zhengzhou, China; ^4^School of Veterinary Medicine, Henan University of Animal Husbandry and Economy, Zhengzhou, China; ^5^School of Food Science and Technology, Henan University of Technology, Zhengzhou, China

**Keywords:** poultry pathogens, *Gallibacterium anatis*, *Leuconostoc mesenteroides* QZ1178, lactic acid bacteria, multidrug resistance

## Abstract

Lactic acid bacteria (LAB) convert carbohydrates into organic acids [mainly lactic acid (LA)], which reportedly have bactericidal activities. *Gallibacterium anatis* is a Gram-negative bacteria which infects birds, and causes significant economic losses. In this study, we investigated the antibacterial activity of the LA producing, *Leuconostoc mesenteroides* QZ1178 from Qula (fermented food), against *G. anatis*, using the Oxford cup method. Our data showed that *L. mesenteroides* QZ1178 inhibited *G. anatis* isolates from different origins; however, *L. mesenteroides* QZ1178 antibacterial activity dropped dramatically at pH 5.5–pH 6. The LA concentration and pH of the liquid broth containing *L. mesenteroides* QZ1178 after 24 h culture was 29 mg/mL and 3.6, respectively. This concentration (29 mg/mL at pH 3.6) and the antibiotic, cefotaxime (minimum inhibitory concentration (MIC) 2.5 μg/mL) effectively inhibited *G. anatis* (GAC026) growth as observed by scanning electron microscopy (SEM) and transmission electron microscopy (TEM). *Gallibacterium anatis* treated with LA exhibited extensive cell surface collapse, increased cell damage, cell membrane disruption, and cytoplasmic leakage, indicative of cell lysis. We suggest *L. mesenteroides* QZ1178 exerts potential antibacterial effects against the poultry pathogen, *G. anatis* via LA.

## Introduction

Lactic acid bacteria (LAB) are used in food preservation, feed fortification, and veterinary medicine (1, 2). They exert bacteriostatic effects by interfering with pathogen cell membrane functions, leading to membrane permeability, loss of cell contents, lysis, and death (3–5). Such antimicrobial characteristics are reportedly due to lactic acid (LA) and phenyllactic acid (6–9). Recently, researchers used LAB strains with desirable properties, to modify gut microbiota and target bacterial pathogens (10–15).

*Gallibacterium anatis* is an opportunistic poultry pathogen residing with the normal microbiota in the respiratory tract and lower genital tract of healthy birds (16, 17). *Gallibacterium anatis* may be divided into two biovars: *G. anatis* haemolytica (GAH) and *G. anatis* anatis (18). GAH causes salpingitis, peritonitis, and respiratory diseases in chickens, leading to decreased egg production, and increased mortality (19, 20). The bacteria was previously isolated from an immunocompromised 26-year-old female with bacteremia and diarrhea, and was speculated to originate from *G. anatis* contaminated food (21).

Widespread multidrug resistance prevents infection treatments with traditional antibiotics (22, 23). As alternatives, LAB strains have been used to inhibit food-borne pathogens, e.g., cell free supernatants (CFS) from *Leuconostoc mesenteroides* (from Kimchi) displayed strong antibacterial effects against both Gram-positive and Gram-negative bacteria (24). Similarly, *L. mesenteroides* (from Melichloro cheese) supernatants effectively inhibited *Streptococcus pyogenes* (25). However, the use of *Leuconostoc mesenteroides* in inhibiting *G. anatis* has not been reported. In this study, we explored *L. mesenteroides* effects toward *G. anatis*, and compared them with existing antibiotics to provide mechanistic insights on infection capabilities.

## Materials and Methods

### Bacterial Strains and Growth Conditions

*Leuconostoc mesenteroides* QZ1178 (*L. m* QZ1178) was previously isolated from Qula, a traditional fermented food from Qinghai Province, China. It was maintained in 25% (v/v) glycerol stocks at −80°C at Henan Key Laboratory of Ion-Beam Bioengineering, Zhengzhou University. *L. mesenteroides* QZ1178 was grown in Man Rogosa Sharpe (MRS; Merck, Darmstadt, Germany) solid media at 30°C for 48 h. In total, 16 *G. anatis biovar haemoltica* strains were used (Table 1). These strains were isolated, identified and preserved in the clinical veterinary laboratory of Henan University of Animal Husbandry and Economy (26). All *G. anatis* strains were cultured on blood agar plates (Bosai Zhengzhou, China) containing brain-heart infusion (Oxoid) agar supplemented with 5% citrated bovine blood in sealed plastic bags at 37°C.

**Table 1:** Inhibition zones of *Leuconostoc mesenteroides* QZ1178 CFS against *Gallibacterium anatis* strains.

***Gallibacterium anatis***	**Location**	**Chicken breed**	**Inhibition zone (mm)**
GAC033 (spleen)	Xinxiang poultry disease hospital	Silky blank-bone chicken	++
GAC034 (liver)	Xinxiang poultry disease hospital	Silky blank-bone chicken	++
GAC038 (Oviduct)	Xinxiang poultry disease hospital	Silky blank-bone chicken	++
GAC040 (intestines)	Xinxiang poultry disease hospital	Silky blank-bone chicken	++
GAC044 (kidneys)	Xinxiang poultry disease hospital	Silky blank-bone chicken	++
GAC105 (In the cleft palate)	Jinan zhangqiu, shandong province	Lohmann pink laying hen	+ + +
GAC157 (cloaca)	Shanghai Pudong New Area	Hyline brown laying hen	++
GAC170 (cloaca)	Jiangxia district, wuhan city, hubei province	Jianghan chicken	++
GAC171 (In the cleft palate)	Jiangxia district, wuhan city, hubei province	Jianghan chicken	+
GAC177 (cloaca)	Jiangxia district, wuhan city, hubei province	Lohmann pink laying hen	++
GAC021 (In the cleft palate)	Tanghe, nanyang, henan	Silky blank-bone chicken	+
GAC032 (Ovary)	Xinxiang poultry disease hospital	Silky blank-bone chicken	+
GAC039 (weasand)	Xinxiang poultry disease hospital	Silky blank-bone chicken	+
GAC046 (spleen)	Shangqiu zhecheng	Silky blank-bone chicken	+
GAC047 (In the cleft palate)	Yangcheng county, jincheng city, Shanxi Province	Hyline brown laying hen	++
GAC048 (cloaca)	Yangcheng county, jincheng city, Shanxi Province	Hyline brown laying hen	+

*The inhibition zone is the external diameter of the cup. Diameter of inhibition zone: –, no inhibition; +, 10–15 mm; ++, 15–20 mm; + + +, 20– 25 mm; n = 3*.

### The Antibacterial Activity of *L. mesenteroides* QZ1178 Against *G. anatis*

The antimicrobial activity of *L. mesenteroides* QZ1178 against *G. anatis* strains was evaluated using the Oxford cup technique (27), with some modification. Briefly, *L. mesenteroides* QZ1178 was inoculated into MRS broth at 10^8^ colony forming units (CFU)/mL and static incubated at 30°C for 24 h. Cultures were centrifuged at 6,000 rpm (4°C) for 10 min, with the CFS collected following filtration (0.22 μm membrane). *G. anatis* (pre-cultured overnight, with an optical density (OD) = 1 at 600 nm) was inoculated onto 5 mL nutrient agar (NA) (50°C) using a 3% inoculant and 5% citrated bovine blood. After solification, sterilized Oxford cups were pressed lightly onto the NA surface, and dropped into 250 μL overnight CFS of *L. mesenteroides* QZ1178. Inhibition zones were measured after a 24 h incubation at 37°C.

### Quantification of Organic Acid Production of *L. mesenteroides* QZ1178

Organic acids, including LA, acetic acid (AA), propionic acid (PA), and butyric acid (BA) were measured using high performance liquid chromatography (HPLC) (Waters 2695 instrument, Waters Co., Ltd., USA; column: Carbomix H-NP10: 8%, 7.8 × 300 mm, Sepax Technologies, Inc., Delaware, USA; detector: DAD, 214 nm; eluent: H_2_SO_4_ 2.5 mmol/L, 0.6 mL/min; temperature: 55°C). The pH was determined using a pH meter (Mettler Toledo MP230; Greifensee, Switzerland).

### Determination of Minimum Inhibitory Concentrations (MIC) for Cefotaxime

MIC determinations were conducted using the tube dilution method (28). Briefly, cefotaxime (Macklin, Shanghai, China) sodium solution was 2-fold serially diluted to 1,280, 640, 320, 160, 80, 40, 20, 10, 5, 2.5, 1.25, 0.625 μg/mL, and 0 μg/mL in tubes. These were inoculated with *G. anatis* (pre-cultured in Muller-Hinton broth, final density = 5.0 × 10^5^ CFU/mL) and incubated at 37°C for 24 h, followed by optical density measurements using a spectrophotometer (UVmini-1240, SHIMADZU, Japan). Five *G. anatis* strains from sick chickens from Xinxiang Poultry Hospital were used. As a negative control, blank sterile nutritional broth was used.

### *Leuconostoc mesenteroides* QZ1178 CFS and Cefotaxime Inhibition of *G. anatis*

*Leuconostoc mesenteroides* QZ1178 was inoculated into liquid MRS medium at 30°C for 24 h. The cell suspension was centrifuged at 6,000 rpm (4°C) and the supernatant collected. Five *G. anatis* isolates from chicken palate, cloaca, and fallopian tube were selected as experimental strains. After preculture on solid media, they were inoculated into LB (containing 10% fetal bovine serum) at 37°C at 180 rpm for 8, 20, and 24 h. *Gallibacterium anatis* strains were then separated into 10 mL tubes, with each containing 4 mL. *L. mesenteroides* QZ1178 CFS was then added at 1:1 (high dose) and 1:10 (low dose) ratios, and cefotaxime added at 1:1 (the concentration was referred to MIC). *G. anatis* strains were cultured for 0, 1, 3, 5, and 7 h before viable cell counting. A bacterial culture without treatment was used as a control. Experiments were performed in triplicate.

### *Leuconostoc mesenteroides* QZ1178 CFS and LA Antibacterial Activities Against *G. anatis* Strains

Antimicrobial activities were tested at different pHs (pH = 4.0, 5.0, 5.5, and 6) using the Oxford cup method. From HPLC, LA was prepared at 29 mg/mL and pH 3.6, and also adjusted with 1 M NaOH to generate different pHs. Approximately 250 μL CFS or LA at adjusted pHs were added to the Oxford cups and incubated at 37°C for 24 h. Bacterial inhibition zones were measured.

### The Inhibitory Effect of Hydrogen Ion on *G. anatis*

MRS medium was used to prepare LA, AA, and hydrochloric acid (HA) solutions. From HPLC, LA was prepared at 29 mg/mL (pH 3.6). AA was prepared at 7 mg/mL (pH 4.5). HA at pH 3.6 was also prepared. Organic acid solutions were added to *G. anatis* cultures at a 1:1 ratio (culture time = 24 h). Untreated *G. anatis* cultures were prepared as controls. Cultures were grown at 37°C for 0, 1, 3, 5, and 7 h before viability measurements. Experiments were performed in triplicate.

### Scanning Electron Microscopy (SEM) and Transmission Electron Microscopy (TEM)

*Gallibacterium anatis* GAC026 and *L. mesenteroides* QZ1178 cultures were grown for 24 h and supernatants collected by centrifugation. LA at 29 mg/mL was prepared using MRS liquid medium at pH 3.6. The cefotaxime concentration was set at the MIC. *L. mesenteroides* QZ1178 CFS, LA, and cefotaxime were added to *G. anatis* cultures at 1:1 ratios, and cells collected at 0, 1, 3, 5, and 7 h at 37°C. Cells were centrifuged at 2,500 rpm for 10 min, washed twice in phosphate-buffered saline (pH 7.2) and resuspended in 2.5% (w/w) glutaraldehyde fixing solution at 4°C for 12 h. Cells were dehydrated in 30, 50, 70, 80, 90, and 100% alcohol for 15 min. After this, they were suspended in 100% ethanol, fixed on a microslide, and sputter-coated with gold under vacuum. They were then examined by SEM (S-4800, HITACHI, Japan). Precipitates were fixed in 4% (w/w) glutaraldehyde for > 4 h. Specimens for TEM (JEM-1400, JEOL Ltd., Japan) were prepared by conventional ultrathin sectioning at the TEM Center of Henan University of Chinese Medicine.

### Statistical Analysis

GLM(General linear model) and one-way ANOVA procedures were performed using the SPSS statistical software package (IBM SPSS Statistics; version 20.0; IBM Corporation, Somer, NY, USA). Duncan's multiple comparisons were used to compare differences between groups. Significant differences were accepted at *P* < 0.05. CFU variations were plotted using Origin software Pro. 9.0.

## Results

### Antimicrobial Activity Using the Oxford Cup Technique

Based on inhibitory zone results (Table 1), the CFS of *L. mesenteroides* QZ1178 displayed potent antimicrobial activities against all *G. anatis* strains. The antibacterial diameter for GAC105 was > 15 mm, and the antibacterial diameters for nine strains were 15–20 mm. Bacteriostasis for six strains was weak (10–15 mm).

### Organic Acid and pH Analysis of CFS

According to HPLC data, the major organic acid in *L. mesenteroides* QZ1178 was LA, followed by AA; the concentrations for both reached 29 and 7 mg/mL, respectively. PA and BA were not detected in *L. mesenteroides* QZ1178 CFS. The pH supernatant declined sharply and remained at pH 3.6 after a 24 h incubation.

### OD Values of Cefotaxime Against Five *G. anatis* Strains (From GAC026 to GAC030)

Table 2 showed the OD values of cefotaxime against five *G. anatis* strains (from GAC026 to GAC030). Like other cephalosporins, cefotaxime is a wide spectrum antibiotic that inhibits the growth of both Gram-negative and Gram-Positive bacteria. Of the tested strains, GAC026 and GAC027 were more sensitive to cefotaxime, whereas GAC028, GAC029, and GAC030 exhibited moderate sensitivity. Increased cefotaxime concentrations led to a marked decrease in *G. anatis* strains viabilily (Table 2). The MIC values for *G. anatis* GAC026 was 2.5 μg/mL when the absorbance was 0.09 at 600 nm, whereas, its viability increased after exposure to 1.25 μg/mL cefotaxime, i.e., the OD was 0.27. Thus, cefotaxime exhibited reasonable antibactericidal activity.

**Table 2:** MIC values and OD values of Cefotaxime antibiotics against five *G. anatis* (GAC026–GAC030).

**I.D. of *Gallibacterium anatis***	**Ceftioxime concentration**						
	**20 μg/mL**	**10 μg/mL**	**5 μg/mL**	**2.5 μg/mL**	**1.25 μg/mL**	**0.625 μg/mL**	**0 μg/mL**
GAC026	0.10 ± 0.01^c^	0.09 ± 0.00^c^	0.09 ± 0.01^c^	0.09 ± 0.01^c^	0.27 ± 0.01^b^	0.26 ± 0.01^b^	0.30 ± 0.01^a^
GAC027	0.07 ± 0.01^b^	0.09 ± 0.01^b^	0.08 ± 0.01^b^	0.11 ± 0.01^b^	0.36 ± 0.02^a^	0.36 ± 0.01^a^	0.37 ± 0.02^a^
GAC028	0.01 ± 0.00^e^	0.01 ± 0.00^e^	0.13 ± 0.01^d^	0.23 ± 0.01^c^	0.36 ± 0.02^b^	0.39 ± 0.01^a^	0.41 ± 0.01^a^
GAC029	0.10 ± 0.01^c^	0.09 ± 0.01^c^	0.09 ± 0.01^c^	0.37 ± 0.01^b^	0.37 ± 0.01^b^	0.61 ± 0.01^a^	0.59 ± 0.02^a^
GAC030	0.01 ± 0^f^	0.17 ± 0.01^e^	0.25 ± 0.01^d^	0.38 ± 0.01^c^	0.55 ± 0.07^a^	0.45 ± 0.01^b^	0.51 ± 0.01^ab^

*Values with different small letters show significant differences in the Ceftioxime concentration treatment (P < 0.05)*.

### *Leuconostoc mesenteroides* QZ1178's CFS and Cefotaxime Inhibited *G. anatis* (GAC026–GAC030) Growth

**Figure 1:**
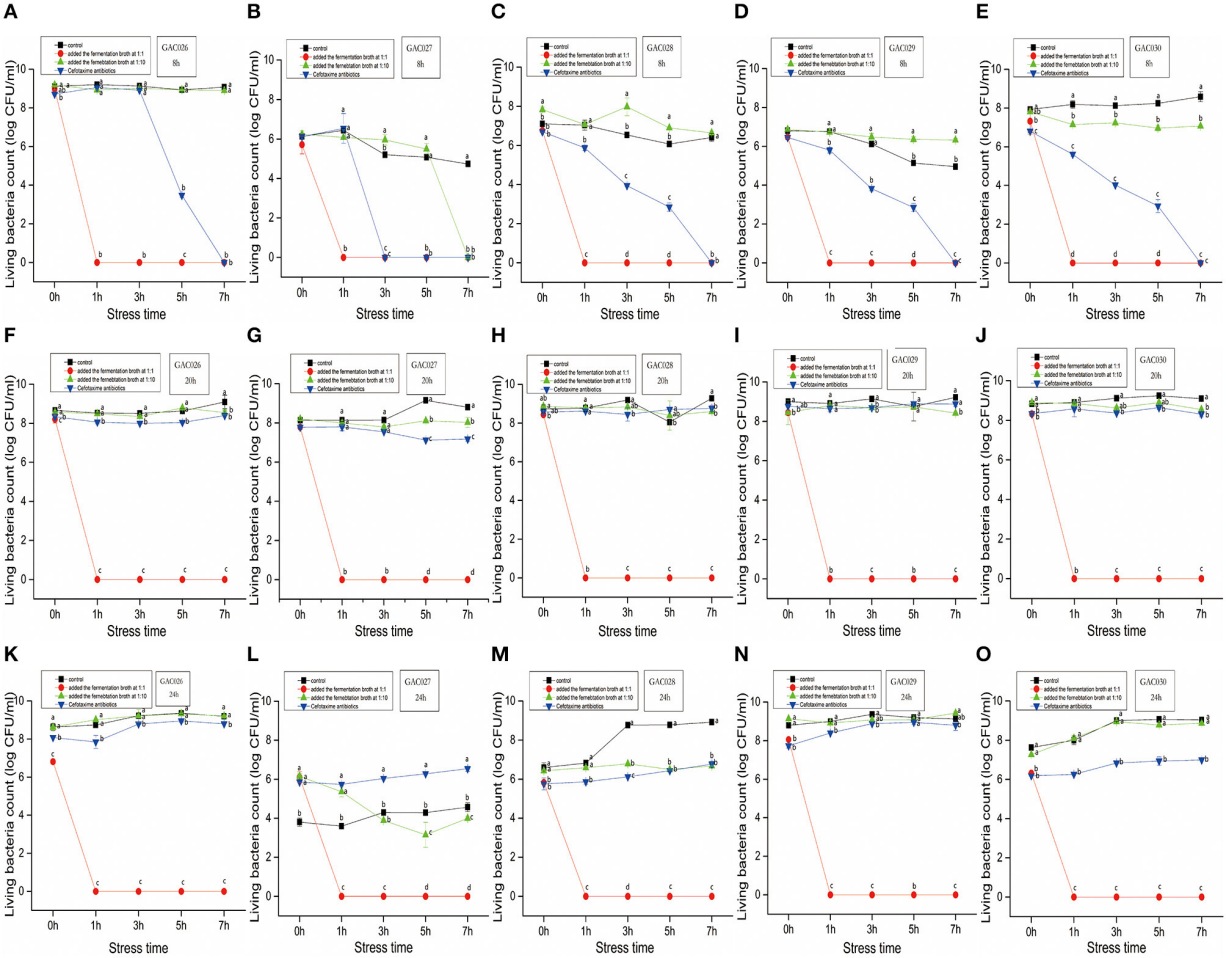
The dynamics by QZ1178's CFS(1:1 vs. 1:10), antibiotics of five *G. anatis*. Effects of growth time (8, 20, and 24 h), treatment (Added the fermentation broth at 1:1 ratio vs. Added the fermentation broth at 1:10 vs. Cefotaxime antibiotics) and stress time (0, 1, 3, 5, and 7 h) on CFU of *Gallibacterium anatis* from GAC026 to GAC030. From **(A–E)**, five strains of CFU with growth time of 8 h were treated with fermentation broth 1:1 and 1:10 and treated with antibiotics. From **(F–J)**, five strains of CFU with growth time of 20 h were treated with fermentation broth 1:1 and 1:10 and treated with antibiotics. From **(K–O)**, five strains of CFU with growth time of 24 h were treated with fermentation broth 1:1 and 1:10 and treated with antibiotics.

The characteristics of five *G. anatis* strains treated by *L. mesenteroides* QZ1178 CFS, antibiotics and control are shown (Figure 1). Most *G. anatis* strains formed biofilms, to different degrees. Initial cell adhesion commenced at 8 h, more at 20 h, with a complete biofilm formed at 24 h. Therefore, 8, 20, and 24 h time points were selected as observation points. After adding a high dose of *L. mesenteroides* QZ1178 fermentation broth, the CFU values for *G. anatis* were significantly reduced (*P* < 0.05) when compared with controls. The CFU rapidly decreased to 0 at 1 h and was still 0 at 7 h. By adding fermentation broth at a high dose, this significantly decreased *G. anatis* strain growth (*P* < 0.05). However, CFU values for *G. anatis* were not decreased after adding a low dose fermentation broth. Organic acids, including short chain fatty acids, LA and formic acid were previously shown to inhibit pathogens in livestock animals, with LA considered a main fermentation product of LAB (29). The highest inhibition rate (at 8, 20, and 24 h) toward *G. anatis* was probably mediated by organic acids in the fermentation broth. At 8 h, cefotaxime addition significantly (*P* < 0.05) reduced the CFU's of *G. anatis* when compared with controls, but no significant decrease was observed at 20 and 24 h, suggesting antibiotic resistance had occurred.

### The Antagonistic Activity of *L. mesenteroides* QZ1178 CFS When Neutralized With Alkali

*Leuconostoc mesenteroides* QZ1178 CFS was evaluated for antibacterial activity against *G. anatis* GAC026 after pH adjustment. The antibacterial activity of *L. mesenteroides* QZ1178 CFS and LA were both lessened at increased pHs. No antibacterial activity was observed at pH 5.5 and 6.0, while the CFS of *L. mesenteroides* QZ1178 showed moderate antibacterial effects against *G. anatis* GAC026 at pH 4 and pH 5 (Table 3). These results suggested LAB mediated acid conditions played a critical role in the antagonistic activity of *L. mesenteroides* QZ1178 CFS.

**Table 3:** Effect of pH on the antagonistic activity of QZ1178's CFS by measuring inhibition zones (mm).

**pH**	**QZ1178's CFS**	**Lactic acid**
4	+ + +	+ + +
5	++	+ + +
5.5	-	-
6	-	-

*The inhibition zone contains the external diameter of the cup. Diameter of inhibition zone: -, no inhibition; ++, 15–20 mm; + + +, 20– 25 mm; n = 3*.

### The Inhibition of *G. anatis* GAC026 Growth Using Different Organic Acids

The effects of LA, AA, and HA on *G. anatis* growth are shown (Table 4). Adding LA, AA, and HA in 1:1 ratios, significantly decreased *G. anatis* GAC026 growth. LA and AA both significantly reduced the CFU *for G. anatis* GAC026 when compared with controls. *G. anatis* CFU decreased gradually after HA was added at 1:1, suggesting strong acids generated no significant inhibitory effects on *G. anatis* GAC026. Cell counts for *G. anatis* GAC026 treated with LA and AA decreased rapidly to 0 h at 1 h, and continued for at least 7 h. This phenomenon was not observed in HA + H_2_O treated *G. anatis* GAC026.

**Table 4:** Effects of different treatment on the growth of *G. anatis*.

**Stress time**	**Treatment**						
	**Control (log CFU/ml)**	**Lactic acid +MRS**	**Lactic acid +H_**2**_O**	**Acetic acid +MRS**	**Acetic acid +H_**2**_O**	**HCL + MRS**	**HCL + MRS**
0 h	9.03 ± 0.05^Aa^	9.05 ± 0.05^Aa^	ND^d^	8.88 ± 0.07^Ab^	4.40 ± 0.11^Ac^	8.94 ± 0.07^Aab^	8.94 ± 0.04 ^Aab^
1 h	9.07 ± 0.15^Aa^	ND^Bd^	ND^d^	3.92 ± 0.08^Bc^	ND^Bd^	3.96 ± 0.16^Bc^	8.79 ± 0.03 ^Bb^
3 h	8.84 ± 0.06^Ba^	ND^Bc^	ND^c^	ND^Cc^	ND^Bc^	ND^Cc^	8.51 ± 0.06^Cb^
5 h	8.78 ± 0.02^Ba^	ND^Bc^	ND^c^	ND^Cc^	ND^Bc^	ND^Cc^	6.53 ± 0.09^Db^
7 h	9.00 ± 0.13^ABa^	ND^Bc^	ND^c^	ND^Cc^	ND^Bc^	ND^Cc^	3.93 ± 0.06^Eb^

*ND, non-detected. Values with different small letters show significant differences in the different treatment. Values with different capital letters show significant differences among treatments in the stress time (P < 0.05)*.

### The Effects of LA, Fermentation Broth, and Cefotaxime on Cell Morphology Using SEM

**Figure 2:**
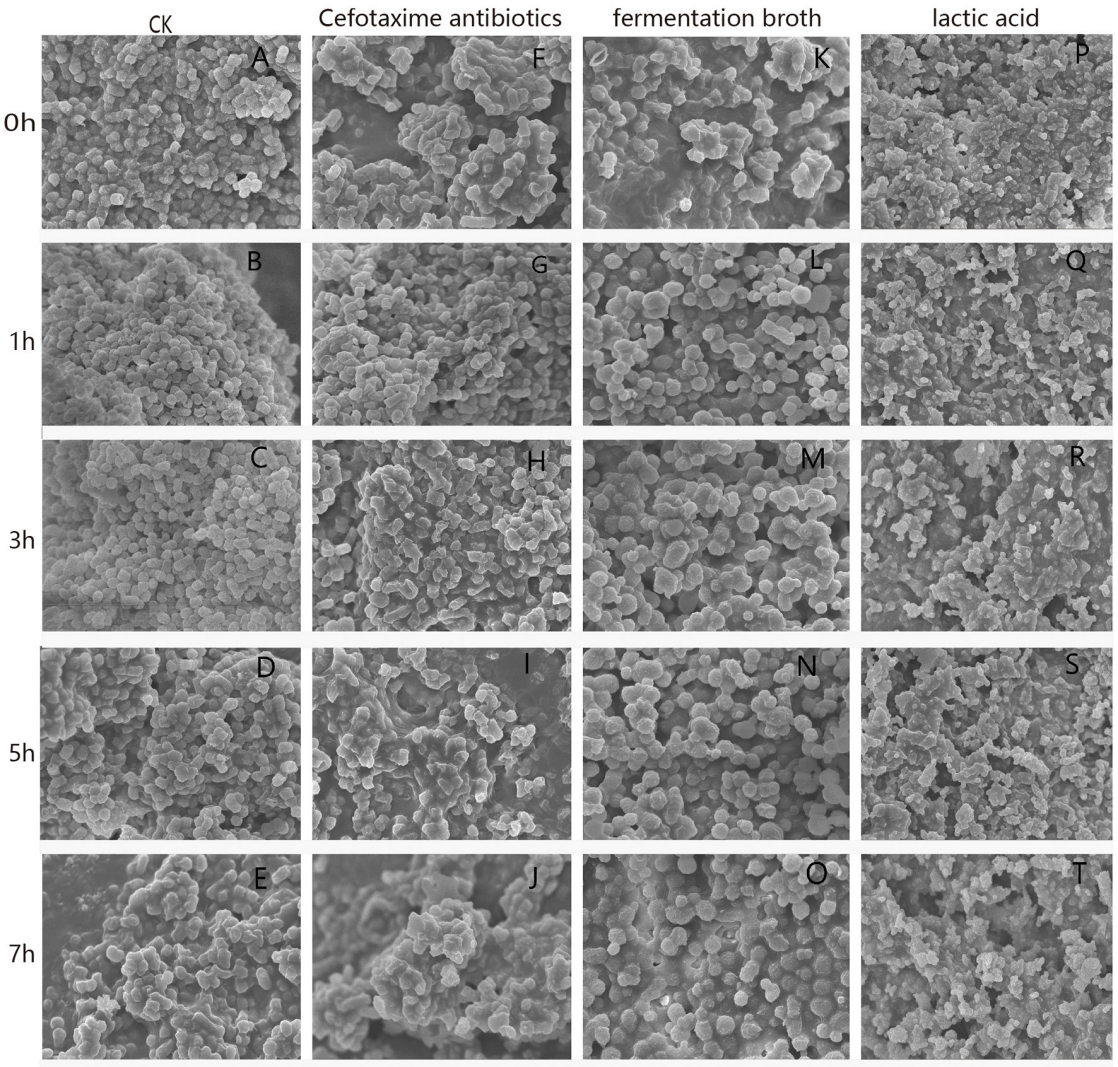
SEM photographs of *G. anatis* GAC026 treated with fermentation broth in 1:1 ratio, cefotaxime antibiotics at MIC and lactic acid at 29 mg/ml for 0, 1, 3, 5, and 7 h: without treatment **(A–E)**, cefotaxime antibiotics at MIC **(F–J)**, fermentation broth in 1:1 ratio **(K–O)**, lactic acid at 29 mg/ml **(P–T)**.

*Gallibacterium anatis* GAC026 cell morphological changes are shown (Figure 2). Untreated cells displayed typical bacilliform and spherical morphology, of uniform size (Figures 2A–E). The cell surface appeared intact and glossy. In contrast, cells treated with fermentation broth at a 1:1 ratio (Figures 2K–O) were irregular, had a wrinkled outer surface, extensive surface collapse, with increased cell damage. Cells treated with LA at 29 mg/mL (Figures 2P–T) showed some pores or localized ruptures in membranes. Intracellular material had leaked from membranes, and form aggregations and adhesions. With prolonged acid stress, GAC026 cells were severely damaged, and most membranes had collapsed and ruptured, generating debris. Such morphological alterations were previously observed for bacteria treated with organic acids (3). Cefotaxime treated *G. anatis* GAC026 cells showed altered cell surface morphology, surface shrinkage, and shrunken walls (Figures 2F–J).

### The Effects of LA, Fermentation Broth, and Cefotaxime on Cell Morphology Using TEM

**Figure 3:**
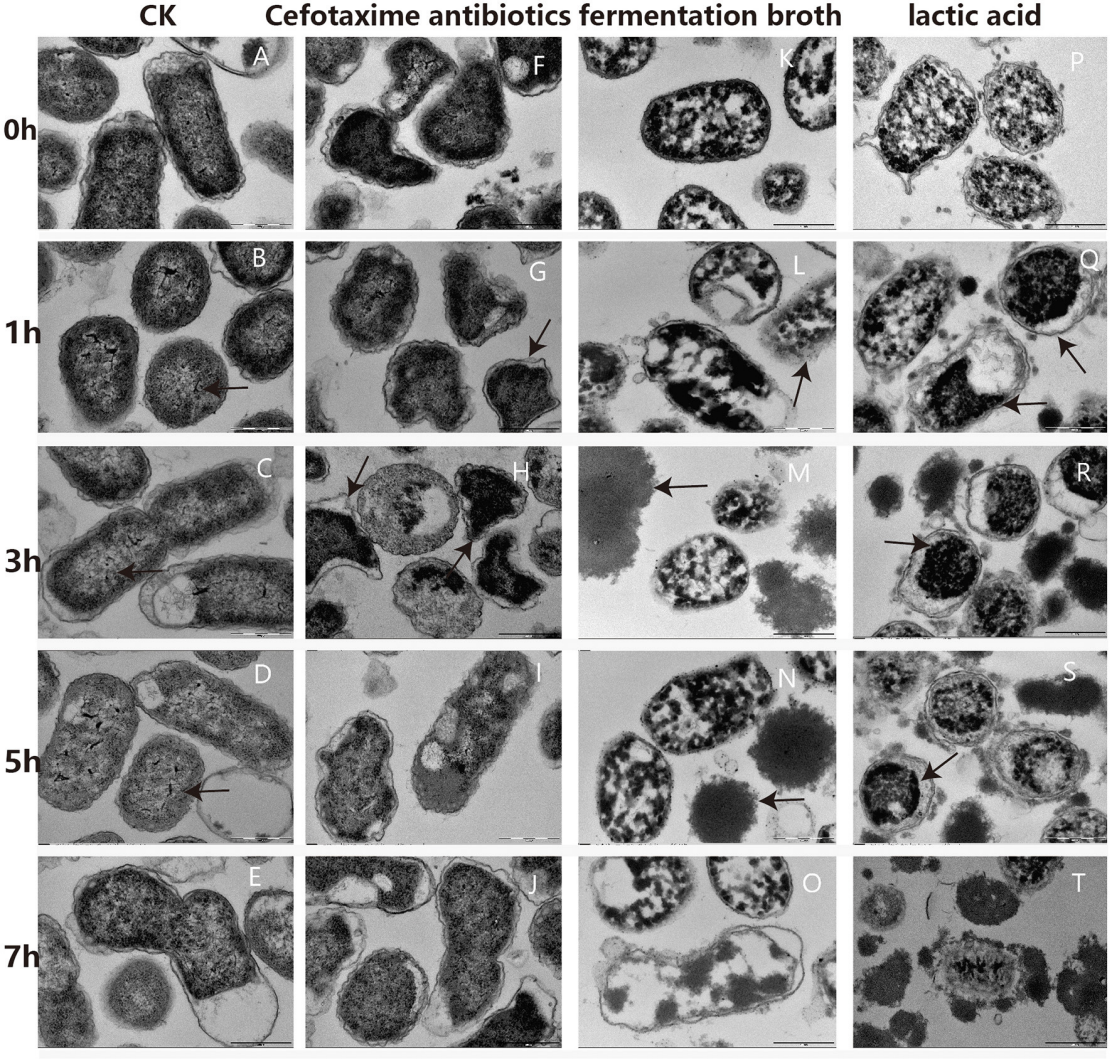
TEM photographs of *G. anatis* GAC026 treated with fermentation broth in 1:1 ratio, cefotaxime antibiotics at MIC and lactic acid at 29 mg/ml for 0, 1, 3, 5, and 7 h: without treatment **(A–E)**, cefotaxime antibiotics at MIC **(F–J)**, fermentation broth in 1:1 ratio **(K–O)**, lactic acid at 29 mg/ml **(P–T)**. The DNA fibrils (arrows in **B–D**). The dividing cell **(E)**. *G. anatis* GAC026 was treated with cefotaxime antibiotics for 0 h **(F)**. Triangular or irregular *G. anatis* GAC026 (arrows in G, H). A big gap between the capsule and the cell wall (arrows in **H**). *G. anatis* GAC026 was treated with cefotaxime antibiotics for 5 h **(I)** and 7 h **(J)**. The cell envelope ruptured (arrows in **L)**. Cells contents without cell walls (arrows in **M,N**). *G. anatis* GAC026 was treated with fementtation broth for 7 h **(O)**. *G. anatis* GAC026 was treated with lactic acid for 0 h **(P)**. The tightly condensed substances or dense granules contracted together (arrows in **Q–S**). *G. anatis* GAC026 was treated with lactic acid for 7 h **(T)**.

Changes in cellular ultrastructure, with and without treatment, in *G. anatis* GAC026 cells were observed by TEM. For the control group, cells exhibited a clear, uniform cytoplasm at 0 h−7 h (Figures 3A–E), including intact DNA fibrils (Figures 3B–D; black arrows) and dividing cells (Figure 3E). However, upon fermentation broth addition for 1 h, some *G. anatis* GAC026 membranes ruptured and cytoplasmic contents leaked (Figure 3K). A clear loss of cell envelope integrity was also observed (Figure 3L).

In general, morphological cell structures exposed to CFS remained unchanged, but membrane structural integrity was severely disrupted in a time-dependent manner. Most cells collapsed or lysed, resulting in death. Cell contents with no cell walls were observed (Figures 3M,N). *G. anatis* GAC026 was treated with fermentation broth for 7 h (Figure 3O). *G. anatis* GAC026 was treated with LA for 0 h (Figure 3P). After LA treatment for 1 h, tightly condensed substances or dense granules contracted together (Figures 3Q–S). *G. anatis* GAC026 was treated with LA for 7 h (Figure 3T). Both fermentation broth and LA changed *G. anatis* GAC026 morphology, leading to cell membrane rupture, and incomplete or total whole cell membrane loss when compared with controls. *G. anatis* GAC026 morphology at 2.5 μg/mL cefotaxime changed from oblong to triangular or irregular shapes (Figures 3F–H). Large gaps between the capsule and cell wall (Figure 3H), and cell wall atrophy or depression were also observed. However, cell contents did not leak (Figures 3I,J).

## Discussion

*Gallibacterium anatis is* a Gram-negative coccobacillus, and commensal inhabitant of the respiratory and reproductive tract of birds. It is an opportunistic pathogen in intensively reared poultry and domestic birds, and has emerged as a multidrug-resistant pathogen causing decreased egg production, salpingitis, peritonitis, and even respiratory tract lesions (20, 30, 31).

Currently, antibiotics are used to treat *G. anatis* infections, however, antibiotic resistance to commonly used therapies, including penicillin, macrolides, and tetracyclines are major concerns, although the pathogen remains sensitive to certain novel antibiotics, e.g., cefotaxime. In agreement, *G. anatis* GAC026 was sensitive to cefotaxime in our study. At 8 h, the antibiotic significantly reduced *G. anatis* when compared with controls, but no significant decreases were observed at 20 and 24 h. Cefotaxime inhibits bacterial cell wall synthesis by competitively inhibiting transpeptidase.

Resistance against cefotaxime increased significantly with the time of growth of *G. anatis* GAC026. Using SEM, *G. anatis* GAC026 in 2.5 μg/mL cefotaxime changed from oblong to triangular or irregular shapes, suggesting cell wall atrophy or depression had occurred, however, cell contents did not leak.

Owing to the emergence of widespread multidrug resistance, traditional antimicrobial drugs are not indicated for this bacterial pathogen. The efficacy of classical vaccines in preventing this disease is limited due to disease antigenic diversity (32). Common *G. anatis* virulence factors include the capsule, outer membrane vesicles, fimbriae, metalloproteases, and biofilm formation, etc. (33–35). Thus, a major problem in combating *G. anatis* is multidrug resistance. For Gram-negative bacteria, typical antimicrobial agents only generate bacteriostatic or bactericidal effects upon cell entry. Outer membranes are composed of asymmetric phospholipid and lipopolysaccharide layers (36, 37). This latter layer forms a tight barrier limiting compound access, including antibiotics (38–40). Additionally, lipopolysaccharide core regions coordinate divalent cations, including Fe^2+^, Mg^2+^, and Ca^2+^ (41). If such cations are removed, lipopolysaccharide molecules are released from outer membranes, exposing underlying phospholipid bilayers, and disrupting outer membrane integrity. Organic acids appear to have unique mechanisms which differ with antibiotics.

Protonated organic acid molecules diffuse directly through cell membranes or pore proteins, to release protons and anions and inhibit cell growth (42, 43). Weak organic acids, such as LA, are more effective than inorganic acids in killing bacteria (44). LABs exert antimicrobial activities by secreting LA, which effectively kill both Gram-negative and Gram-positive pathogens (45). These molecules lower the surrounding pH which stresses cells. Equally, protonated LAs permeabilize cell membranes and enter the cytoplasm, where they dissociate and lower the internal pH (Gallmann, 2003). Theoretically, undissociated elements of acid molecules are primarily responsible for antimicrobial activity, while their effectiveness at a given pH depends to a large extent on the dissociation constant (pKa) of the acid. However, fully dissociated “strong” acids (e.g., HCl) do not contain undissociated elements of the acid molecule, and “strong” acids can only affect bacterial cells by changing the pH (proton concentration) (46). As observed here, LA was more effective than strong acids like HCl in inhibiting *G. anatis* growth at pH 3.6, due to its dissociation and unique pKa. Our TEM data suggested that exposure to 1:1 ratios of fermentation broth and LA of the same concentration, inhibited *G. anatis* growth via cell membrane destruction. Thus, our study extends the knowledge on soluble metabolites responsible for CFS effects from LAB. We showed that acidic supernatants at pH 5.5 displayed no inhibitory activities against pathogenic bacteria, suggesting any inhibitory effects were probably due to organic acids such as LA.

LAB can modify host gastrointestinal microbiota, and prevent respiratory and genitourinary infections (47). This study only confirmed the *in vitro* antibacterial effect of *L. mesenteroides* QZ1178 by generating the acid microenvironment. Further research is required to assess whether *L. mesenteroides* QZ1178 or its metabolites interact with the immune system to enhance defense capabilities against systematic or local infections.

## Conclusions

We compared changes in *G. anatis* treated with *L. mesenteroides* QZ1178 CFS, cefotaxime, and LA. *G. anatis* growth was significantly inhibited after a 1:1 ratio of fermentation broth was added to cells, whose effect was compromised when the acid was neutralized. From our *in vitro* work, we concluded *L. mesenteroides* QZ1178 was a probiotic which may inhibit bird gut microbiota pathogens via LA generation.

## Data Availability Statement

The original contributions presented in the study are included in the article/supplementary material, further inquiries can be directed to the corresponding author/s.

## Author Contributions

HZ, HH, XW, SZ, YL, HL, and GQ designed and did experiments. ZT superivised the project. HZ drafted and finalized the manuscript. All authors contributed to the article and approved the submitted version.

## Conflict of Interest

The authors declare that the research was conducted in the absence of any commercial or financial relationships that could be construed as a potential conflict of interest.
